# Detecting Topic and Sentiment Trends in Physician Rating Websites: Analysis of Online Reviews Using 3-Wave Datasets

**DOI:** 10.3390/ijerph18094743

**Published:** 2021-04-29

**Authors:** Adnan Muhammad Shah, Rizwan Ali Naqvi, Ok-Ran Jeong

**Affiliations:** 1 Department of Information Technology, University of Sialkot, Sialkot 51310, Pakistan; 2 Department of Unmanned Vehicle Engineering, Sejong University, Seoul 05006, Korea; rizwanali@sejong.ac.kr; 3 School of Computing, Gachon University, Seongnam 1342, Korea

**Keywords:** online reviews, text mining, topic modeling, sentiment analysis, COVID-19, discrete emotions

## Abstract

(1) Background: Physician rating websites (PRWs) are a rich resource of information where individuals learn other people response to various health problems. The current study aims to investigate and analyze the people top concerns and sentiment dynamics expressed in physician online reviews (PORs). (2) Methods: Text data were collected from four U.S.-based PRWs during the three time periods of 2018, 2019 and 2020. Based on the dynamic topic modeling, hot topics related to different aspects of healthcare were identified. Following the hybrid approach of aspect-based sentiment analysis, the social network of prevailing topics was also analyzed whether people expressed positive, neutral or negative sentiments in PORs. (3) Results: The study identified 30 dominant topics across three different stages which lead toward four key findings. First, topics discussed in Stage III were quite different from the earlier two stages due to the COVID-19 outbreak. Second, based on the keyword co-occurrence analysis, the most prevalent keywords in all three stages were related to the treatment, questions asked by patients, communication problem, patients’ feelings toward the hospital environment, disease symptoms, time spend with patients and different issues related to the COVID-19 (i.e., pneumonia, death, spread and cases). Third, topics related to the provider service quality, hospital servicescape and treatment cost were the most dominant topics in Stages I and II, while the quality of online information regarding COVID-19 and government countermeasures were the most dominant topics in Stage III. Fourth, when zooming into the topic-based sentiments analysis, hot topics in Stage I were mostly positive (joy be the dominant emotion), then negative (disgust be the dominant emotion) in Stage II. Furthermore, sentiments in the initial period of Stage III (COVID-19) were negative (anger be the dominant emotion), then transformed into positive (trust be the dominant emotion) later. The findings also revealed that the proposed method outperformed the conventional machine learning models in analyzing topic and sentiment dynamics expressed in PRWs. (4) Conclusions: Methodologically, this research demonstrates the ability and importance of computational techniques for analyzing large corpora of text and complementing conventional social science approaches.

## 1. Introduction

Coronavirus (COVID-19) disease is a modern infectious pathogen, transmitted primarily through respiratory droplets and close contacts, and is infectious to humans as a whole [1]. The virus originated from Wuhan, China, in late December 2019. The infection has spread rapidly across more than 100 countries globally, including the United States (U.S.), Japan and Korea. The first confirmed case of the infection was diagnosed in the U.S. on 20 January 2020 [2]. The spread of the virus exponentially increased in the U.S. over the next two months, with major outbreaks took place in urban areas such as New York, Detroit, New Orleans and the San Francisco Bay Area [3]. At the time of this writing, 8.26 million confirmed cases with 220 K deaths were reported in the U.S. [4], leaving health officials scrambling in search of ways to avoid the disease and to reduce the detrimental impact on the citizen’s health and economy.

Following the COVID-19 crisis, it sparked a dynamic network of public opinions. Rumors, abuse and other special factors are currently limiting the beneficial impact of social media in society. It is important to examine online public opinion because if it is not actively driven, it will trigger a crisis in public opinion [5,6]. Through analyzing texts from real-time media, the opinions and sentiments of the subconscious-minded individuals are gathered in order to understand the true cognitive ideas of people [7]. Patients post physician online reviews (PORs) on PRWs, whereas other patients follow them to evaluate a physician quality [8]. Since the utilization of social media is so common among the public, the unique advantages would make it easier for the public to access and communicate information, resulting in an effective response to the crisis [9].

Patients in healthcare market are usually unsure of the consistency of their physicians due to the information asymmetry about service measures, such as diagnostic accuracy, time spent with patients and waiting time. Over the last few years, researchers have made significant efforts to enhance the healthcare quality, and more recent research has explored the efficiency of individual physicians [10]. Recently, patients are increasingly relying on PORs to supplement their limited knowledge. With the emergence of social media usage, patients can locate doctors more quickly, read feedback from other patients, see available appointment hours and book appointments immediately [11]. This study highlights some statistics to get a sense of the fast-growing online health market. First, a profound shift like the patients’ knowledge needs now exists because patients are asking for online information for a wider understanding of health status and treatments. A recent survey showed that nearly three-quarters (72%) of people look for PORs before their initial search for finding a good doctor [12]. Moreover, a recent investigation by Grand View Research Inc. reported that the global digital health market is projected to hit $509.2 billion in value by 2025 [13].

While past pandemics emphasize the critical nature of studying infectious disease-related social media content [14,15,16,17], there is a special significance and urgency to studying COVID-19-related social media content [18]. This study anticipates that social media will play an even greater role in disseminating knowledge about COVID-19. Several studies have shown that social media can be a valuable source of data for predicting outbreaks and recognizing public perceptions and behaviors during a crisis, which can help with crisis communication and health promotion messaging [19,20]. With the emergence of online communication technologies, analyzing social media data helps understand people concerns, awareness and public attention towards the disease [14,21].

Analyzing internet data showing individuals voluntarily views and knowledge on COVID-19 and other healthcare aspects, such as specific PORs from physician rating websites (PRWs), offers a valuable opportunity to recognize and track public perceptions about COVID-19 and the diffusion of relevant statistics on the internet. Given the need to control rumors and observe public sentiment and conduct in the sense of mass infectious diseases and the significance of internet sentiment during the crisis, online data analysis has great repercussions on creating an adequate provision of knowledge and efficient health policies. These PORs provide a wealth of knowledge about different topics that health professionals and researchers cannot assess using a conventional survey questionnaire. Therefore, moving away from the conventional survey methods of data collection, analyzing online data is the gold mines contributing to formulating public health policies [22].

Information sources differ in terms of their credibility, comprehensiveness and testability. Social networks and other Internet channels do not require industrial legislation [1]. In an earlier investigation regarding the analysis of Twitter data, it has been found that 1/4th of the COVID-19 tweets showed misinformation. It has also been indicated that the amount of COVID-19 data and user-generated content on these sites includes a large proportion of rumors, baseless claims, and disinformation, which has also proven to be a challenge for social media outlets [23]. While researchers used these platforms as tools to analyze user-generated content, the speed and extensive scope of such platforms add a digital print to data analytics research on an infectious disease outbreak.

Several attempts to analyze social media data to measure public perceptions were made to look at various infectious disease outbreaks such as Ebola [24], Zika [14,21], H7N9 [25], dengue fever [15] and the Middle East respiratory syndrome (MERS) [17]. Web data analysis is incredibly useful in the first wave of the pandemic. In the first wave of a new pandemic, health officials may have a shortage of sufficient health protocols, and individuals may not find reliable information from other platforms. Due to this scenario, uncertain information on the internet may impact individuals more. To prevent the improper distribution of misleading or excessive fear, web-based data monitoring in the first wave of the pandemic is thus significant [22].

It is necessary to measure individual responses to pandemics and infodemics (information overload). COVID-19 pandemic’s effect means that it is critically important to consider public opinions and how it affects individual behaviors. Failure to do so increases both the time and contingency cost. Web information is also a critical source to understand individuals’ reactions and concerns, and this information can be useful to healthcare organizations in developing interventions. However, relatively little is known about individuals’ reactions to both pre-COVID-19 and during the first wave of COVID-19 disease outbreak on PRWs, especially in the U.S. context. Analysis of real data using public responses could contribute to earlier identification of shifts in public preferences, variations in well-being and public health interventions concerning the COVID-19 pandemic, both of which affect the individuals and the community-level health. Such data can allow policymakers, health practitioners and the general public to recognize and deal with primary issues of concern.

Since the first wave of the COVID-19 outbreak, RateMDs, Healthgrades, Vitals and Yelp, the main PRWs in the U.S., have been frequently involved for the public to discuss the outbreak. Consequently, the community’s response to the COVID-19 outbreak in these PRWs is instantly important to administrative and non-administrative units. Findings from this investigation can provide insights on trends related to the pre-COVID-19 and during COVID-19 pandemic, evaluate variations in discussion topics over time and unnoticed sentiments intensity and trends related to the pre-COVID and during COVID-19 pandemic. The findings can help guide federal and state agencies, business organizations, education institutions, health care institutions and individuals as they search the pandemic-related information.

This study explores the top public concerns over pre-COVID-19 and in the first wave of the outbreak using data from the four U.S.-based PRWs, as these allow users to post online reviews regarding their treatment experiences. This study collected 161,599 PORs for approximately 11,422 physicians containing comments posted on four PRWs in the pre-COVID-19 stage (between March and August of 2018 and March and August of 2019) and during the first wave of the COVID-19 outbreak (between March and August of 2020) to analyze the features of the public concerns and the adequacy of the knowledge circulated online. To sum up, this research addresses the following research questions:

(1) What are the prime concerns expressed in PORs during pre-COVID-19 and the first wave of the COVID-19 disease outbreak?
(2) How do the hot topic keywords differ across the three stages?
(3) How do the topics observe in PORs change by time and COVID-19?
(4) How do PRWs users react to three different stages?

To the best of authors’ knowledge, this is the first research that uses dynamic topic modeling to analyze healthcare big data that span such as long period of time. Based on social media analytics research using various ML algorithms, this study demonstrates how dynamic topic modeling is useful for analyzing healthcare debates because it allows us to examine them from three different perspectives. First, this research uses topic scores to track hot topics of discussion over time; second, this research looked at how the topics of discussion in PRWs changes over time, and third this research identifies the dominant emotions in the topics that are indicative of a specific time span.

Social media analytics research using healthcare big data would enable health authorities and governments to learn about their services and their communications strategies from critical perspectives. Such strategies should be incorporated to enable a more productive public health response to prevent crisis during current and future potential pandemics. The discussion topics steadily transformed from the general healthcare discussion to the possible effects of the outbreak over time, and they continued to attract attention for a long time. The study results indicate that policymakers, governments and local communities use healthcare big data to predict pandemic disease risk and evaluate public reaction and behavior, as well as to develop strategies to avoid the dissemination of negative emotions on social networks and to mitigate their negative impact if pandemics occur again.

The remaining part of this study is organized as follows: Section 2 contains the related work and Section 3 explains the methods employed, followed by results in Section 4. Section 5 discusses the results obtained, details the limitations and recommends future studies. Finally, Section 6 provides the conclusion of the study.

## 2. Related Work

Topic modeling and opinion mining have been extensively used to highlight problems and individuals’ opinions regarding public health. The current study will only present several examples on COVID-19. Jang, et al. [26] applied topic modeling to investigate COVID-19–related themes using Twitter data in North America. Zhao, Cheng, Yu and Xu [1] identified hot topics using Sina Microblog data related to COVID-19 in China. Doogan, et al. [27] used a hybrid method that combines computational and qualitative approaches to characterize public response toward non-pharmaceutical interventions across six countries (Australia, Ireland, Canada, New Zealand, the United Kingdom and the U.S.). Doogan, Buntine, Linger and Brunt [27] also used topic modeling to examine COVID-19 related themes from the Twitter data and to analyze the sentiments toward COVID-19 in the U.S. Based on the econometric analysis and topic modeling, Gozzi, et al. [28] analyzed the online public response to the COVID-19 crisis in four countries: Italy, the U.S., Canada and the United Kingdom. Saleh, et al. [29] performed topic modeling, sentiment and emotion analysis to identify the major topics discussed in the Twitter hashtag sample #socialdistancing and #stayathome. Using topic modeling, Mengying, et al. [30] extract and retain the most useful words from the COVID-19 Open Research Dataset. Xue, et al. [31] used English tweets from different countries and applied topic modeling to abstract trends in public opinions on Twitter. Chen, et al. [32] also applied topic modeling and opinion analysis to verify whether “Chinese Virus” and “COVID-19” are substitutable. Apart from these studies, several other studies also applied topic modeling to identify different trends in public opinion toward COVID-19 [6,18,22,33,34,35,36,37,38,39,40].

This paper is unique from other investigations because authors analyzed PORs datasets from four U.S.-based digital platforms. The healthcare agencies in the U.S. regulate these platforms. There is a systematic monitoring mechanism whenever people post online reviews about the physicians’ quality of care. PRWs owners monitor this content. A POR containing abusive content, disinformation, false claims and rumors cannot be posted on PRWs. The platform filtering system automatically deletes this content before it becomes visible to the public.

Moving away from the traditional topic modeling approach, this study employed a hybrid approach using the unsupervised technique of dynamic topic modeling and aspect-based sentiment analysis (ABSA) to obtain domain-dependent aspect category detection and opinion terms.

## 3. Methods

This study employed machine learning (ML) techniques to identify key topics and terms related to various aspects of healthcare. This study followed a comprehensive PRWs data mining strategy that included data preparation and analysis. Data preparation included three phases: (1) sampling, (2) data collection and (3) pre-processing the raw data. After pre-processing the raw dataset, researchers moved on to the data analysis stage, which included: (1) unsupervised ML (DTM), (2) qualitative analysis (topic labeling) and (3) sentiment analysis (ABSA). Each POR posted on PRWs serves as the study unit of analysis. The broader selection criterion of the study was well suited to the goal of determining how the trends in PORs debate had changed over time. Researchers would lose valuable details regarding healthcare in various time periods if they just looked at PORs from a single time span.

### 3.1. Data Collection

This paper evaluated trends in the U.S’ public conversations using PORs data. Data collection was started on September 02, 2020, approximately covers the latest weeks in the PRWs database. Using the Python network spider, the researchers crawled reviews from those U.S states which are most affected by the disease outbreak. For the analysis, this study selects PORs from four U.S.-based PRWs (RateMDs, Healthgrades, Vitals and Yelp). On 15 August 2020, the authors searched the Web traffic ranking for these websites in the U.S. by Alexa (www.alexa.com accessed on 15 August 2020) and Google Trends and found the highest traffic ranking for these sites.

These sites provided the largest online healthcare services in the U.S. The conversational data on these sites are also accessible for free using different automated methods independent of personal information collection and user approval. The study final sample contained 161,599 PORs for approximately 11,422 physicians, including patients’ comments posted on four PRWs in the pre-COVID-19 stage (between March and August of 2018 and between March and August 2019) and during the first wave of the COVID-19 outbreak (between March and August of 2020).

### 3.2. Data Preprocessing

The authors performed several preprocessing steps to clean the raw data to ensure quality, noise reduction and incoherence. The preprocessing plan was performed in the following steps using Python programming language:

(1) All reviews were converted to lower-case text.
(2) The Python package NLTK [41] based standard approach was used for tokenization, lemmatization, part-of-speech tagging and stop word removal from PORs.
(3) Special characters, symbols, URL’s, punctuations, numbers, words occurrence fewer than 10 times in the corpus, redundant words in the dataset such as cardinal numbers, prepositions, pronouns, etc., were removed. This content did not contribute to the review analysis.
(4) Using the langid package [42], all non-English characters (non-ASCII characters) were removed from the corpus because the analysis focused on the English content of PORs.
(5) All white spaces were removed whenever necessary to create tokens.
(6) All repeated words were converted to their base form. For example, “sooooo happy” was converted to “so happy”.
(7) All unigrams and bigrams were retained in the dataset. In this way, 2 objects, such as secondary effects, were preserved regularly in contiguous sequences. After preprocessing 152,729 PORs for 10,232 physicians met the criteria for analysis.

### 3.3. Topic Modeling

Topic models are unsupervised algorithms for ML to identify latent topics in large text collections. They are based on the premise that co-occurrences of words in a document collection signify semantic proximity. Topic models learn a series of topics, which include various combinations of co-occurring terms in the documents of a corpus on the basis of their frequency. Distributions of words were used to classify topics mathematically. The documents were weighted differently according to their topics (so-called topic scores). Thus, topic models transformed high-frequency terms into two lower-dimensional distributions: the distribution of terms within topics, as well as the distribution of topics within documents [43]. Topic modeling has also been recognized as the most powerful method for textual data classification, clustering and retrieval, and it has been the focus of extensive literature investigation. Many frameworks for topic analysis are extensions of prominent algorithms which are seen as cutting edge for topical modeling.

Latent Dirichlet allocation (LDA) [43] is known as the basic module for probabilistic topic modeling. LDA is a sound technique for qualitative data analysis of large datasets due to its precise topic interpretations. However, LDA overlooked the temporal feature that exists in several record collections. Consider a topic about LDA’s physician healthcare quality in a corpus of PORs from the past few years; the most probable keywords are “trust”, “confidence” and “blessing”, etc. However, the model considers a document from 2018 and 2019 the same as one from 2020 regarding the term probabilities, so it is inadequate to learn that the terms “COVID” and “Corona” for instance, were almost missing in the context of healthcare until relatively recently.

For this research, the authors chose dynamic topic modeling (DTM) [44] as it allows to consistently and integrated model topics to change over time. DTM is an LDA variant that considers changes in topics over discrete time stages. As with other approaches to topic modeling, DTM uses a bag of words, which is to say that a text reduced to word counts and thus disregards the order of terms. The DTM is constructed using a generative statistical model that is adapted to the data provided the weights of terms are derived from the Dirichlet distributions in the topic and the topics in the documents. DTMs [45,46,47], as suggested by Blei and Lafferty [45], addressed the problem by expanding the LDA concept to allow thematic representations to unfold over a fixed period such as years. In particular, each period’s documents are modeled with a topic model of a similar dimension, and each topic in period *t* originates from a respective topic in a time period *t*-1. In DTM, each document is allocated to a specific time bin. The hyperparameter σ (top_chain_var) indicates the variance of the potential shift. This enables the model to track changes in word use over time and thus the creation of a topic.

The LDA generational mechanism remained more or less the same apart from the significant distinction that the overall topic and terms distribution varied in terms of time. Specifically, the parameters for these distributions “originated” at each period by being drawn from distributions centered on the respective values from the preceding time period. The outcome was a sequence of LDA-like topic models that were tied together sequentially. Therefore, a topic that a DTM learns is a sequence of associated distributions over terms. Finally, DTM was applied to the preprocessed PORs datasets.

**Figure 1:**
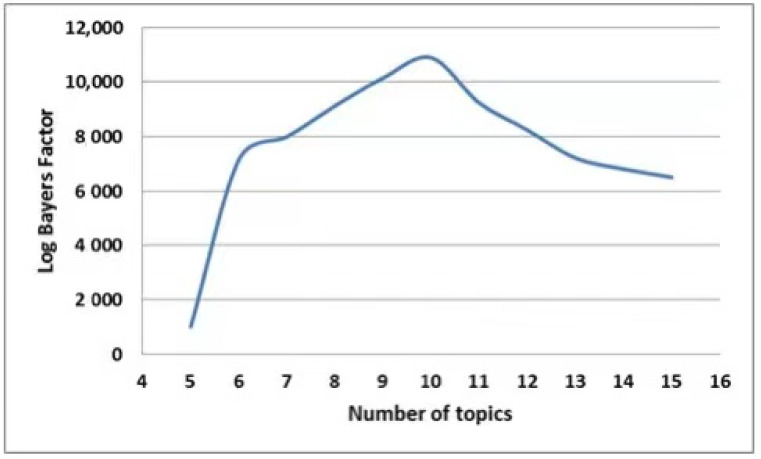
Selection of the number of topics using the log Bayes factor.

In order to find out the number of topics to be used for analysis, we used the log Bayes factor [48]. Log Bayes factor reflects the probability ratio used for model evaluations. It allows for picking the most suitable number of topics by measuring it against a 1-topic model for a number of topics. The log Bayes factor topic selection method yielded 10 as the most interpretable topics (see Figure 1), so the authors decided on the DTM. This can undoubtedly be strengthened by further studies and a thorough quantitative assessment.

Employing Gensim’s Python wrapper to the original DTM Python code, understanding DTM parameters is simple, though slow. The study model took 6 h to run on a computer Windows 10 platform with an i7 processor and 64 GB RAM. Following the topics’ collection, the authors checked the top 10 terms associated with each topic, and a label was drawn for each topic.

In addition, ROST CM6.0 software was used to compute word frequency statistics for the 4 platforms hot topics related to the 3 stages. Finally, the frequencies of keywords corresponding to the hot search topics on 4 PRWs were extracted. For the keywords visualization, the VOSviewer software [49] was employed to construct a visual information map of keyword co-occurrence analysis, keeping in view the keyword co-occurrence frequency as the weight.

### 3.4. Topic Dynamics

To analyze the prevalence of health-related topics across all comments, the authors tracked the topic dynamics over time. The fundamental analysis of a document-to-topic distribution provided by the model was based on the analysis of the estimates θ. To measure the degree of changing dynamics, the authors divided PORs into 6-month time slice for the year 2018, 2019 and 2020, for instance, Mary, April, May, June, July and August. Next, the authors calculated a mean θ vector for PORs in each time slice as performed in [27]. Following the mean θ vector for each time slice, the authors drew graphs of health-related topics over time.

### 3.5. Sentiment Analysis

Understanding public reactions to the healthcare quality is essential to health policymakers because it tells how health policy-makers should frame their health messaging. The authors employed ABSA to capture the sentiment expressed in PORs towards important aspects of healthcare quality. ABSA enabled the exploration of sentiment values of various terms and aspects within a phrase. For example, the doctor treated his patients politely, but the treatment cost was too high, and public transport could not approach his clinic location. A sentence-level opinion mining might not precisely compute the sentiment value due to the presence of multiple positive and negative sentiments. ABSA showed a positive polarity with doctor attitude and a negative sentiment concerning the treatment’s cost and clinic location. In the current study, the authors performed ABSA under the umbrella of sentic computing as performed in [50]. Aspects can include public health interventions or issues associated with healthcare service quality such as “infection”, “competent” and “isolation”. The authors examined public sentiment (positive/negative) toward these aspects.

Sentic computing is a multi-disciplinary concept-level approach that focuses on artificial intelligence and Semantic Web procedures for adaptation and content awareness. Moving away from conventional ML approaches, sentic computing focused on hybrid approach between statistical methods and knowledge-based polarity detection. The method explored concepts rather than words and used those areas of knowledge to extract the context of unclear texts [50]. Relying on a three-layer configuration (i.e., syntactic, semantic and pragmatic), sentic computing tackled the subject of concept ambiguity and other issues generally associated with statistical methods [51]. SenticNet, under the umbrella of sentic computing [52] is an open-source lexical resource for concept-level sentiment analysis, maneuvering both artificial intelligence (AI) and semantic web practices to extract and represent polarities embodied in common-sense concepts in a semanticized structure. People can quickly grasp explicit knowledge in the form of common-sense knowledge but usually lack implicit knowledge. In AI, common-sense knowledge refers to implicit knowledge; humans depend on this category for experiences such as object relevance in the real world, human goals in everyday life, and the sentimental material of diverse occasions [53]. For instance, “to achieve an objective”, “to experience a bad sense” and “to celebrate a special occasion”; these expressions are also used as a means of expressing opinions and handling various events. SenticNet provides us with this type of information [54]. SenticNet’s efficacy and implementation have been extensively studied in IS research [52,54]. The latest version of SenticNet (i.e., SenticNet6) associated a polarity value to about 200,000 common-sense knowledge concepts by coding a similarity algorithm and clustering related words together [54].

Polarity detection using SenticNet determined whether the PORs expressed positive or negative sentiments, along with the strength of sentiments (i.e., aggregate score or opinion score). SenticNet was used to determine the input sentence overall sentiment polarity using a concept-level sentiment analysis method. The framework employed a hybrid approach that integrates semantic structures, conceptual dependency illustrations and ML to deal with concept-driven sentiment analysis. The system processed information across multiple segments before extracting a polarity. The sentence was preprocessed and tickled into a bag of concepts by a Sentic Parser before being inspected against the SenticNet concept collection [54]. Suppose that concepts or sentic patterns do not appear within the concept database; in that case, the sentence is treated as a bag of words and processed with the ML module, a state-of-the-art method for hierarchical relationships to compute sentence polarity.

Sentic patterns refer to the linguistic patterns that allow opinions to flow from concept to concept on the basis of the input sentence dependency, thus producing a binary (positive or negative) polarity value that represents the sentiment of the presenter [55]. If these patterns are included in the input sentence, the input sentence’s sentiment will be sorted concept-wise using dependency-based rules and produce an overall polarity value. If sentic patterns are not matched, the input sentence will be processed through hybrid Sentic-LSTM [56], a sentiment-augmented LSTM on a bag of concepts, to obtain a sentiment polarity value. In addition, the emotions of PORs were analyzed further using the algorithm CrystalFeel (www.crystalfeel.socialanalyticsplus.net accessed on 15 August 2020, [57]), a high-accuracy sentiment analytic tool.

### 3.6. Hybrid Sentic-LSTM

This paper incorporated ideas from the previous work of Ma, et al. [56], who suggested a simplified form of Sentic-LSTM, a combination of a hybrid of LSTM and recurrent additive network. The inspiration behind the study Sentic LSTM [58] used external knowledge to produce the hidden outputs and regulate the information flow. In comparison to the Sentic-LSTM, this version of the Sentic-LSTM could provide concept-level feedback to the recurrent relation and retain a smaller number of parameters.

The proposed neural network framework of the study includes two components: the sequence encoder (hybrid Sentic-LSTM) and hierarchical attention components. Following a sentence s with input words = {w1, w2, …, wL}, a search operation is first implemented to transform input words into word embeddings {v1, v2, …, vL}, where L reflects the length of the sentence. The sequence encoder transforms the word embedding into a sequence of hidden outputs, based on which the attention model is constructed. Target level attention includes the hidden outputs of target expression as a vector representation and automatically encodes the salience of every word. At the second step, the target embedding and aspect embedding are then fed as queries to a sentence-level attention model that transforms the entire sentence into a vector. Finally, a softmax layer is employed for mapping the sentence vector to an output label that mutually reflects its opinion polarity and association of an aspect.

### 3.7. Models Classification

The DTM enables us to reconstruct how topics evolve over time. To train and detect topics in documents, LDA topic models depend solely on the occurrence of words. Non-negative matrix factorization employs the factor analysis approach to give a lower weighting to terms with low coherence. Latent semantic analysis is an effective way of analyzing the text and hidden topics by considering the background of the text.

For the ABSA, the authors compare the performance of the proposed approach to other sentiment analysis approaches proposed to emphasize the technical contribution. The training set included 80,244 PORs and the testing set included 32,488 PORs. This study evaluated and reported the performance of the proposed approach with the other models used for topic modeling and sentiment analysis using evaluation metrics, that is, accuracy and recall score.

(1) $$\mathrm{Accuracy}=\frac{\mathrm{number}\;\mathrm{of}\;\mathrm{true}\;\mathrm{positives}}{\mathrm{total}\;\mathrm{number}\;\mathrm{of}\;\mathrm{instances}}$$

(2) $$\mathrm{Recall}=\frac{\mathrm{True}\;\mathrm{positive}}{\mathrm{True}\;\mathrm{positive}+\mathrm{False}\;\mathrm{negative}}$$

## 4. Results

### 4.1. Topics and their Corresponding Keywords Related to Three Stages on PRWs

The entire set of top 10 topics and their corresponding keywords for the three stages of the people reaction in PORs are listed in Appendix A (Table A1). The most prevalent topics in Stage I was related to different aspects of healthcare providers (i.e., doctor competence, doctor attitude, communication (listen and explain), friendly staff and doctor value). The second most dominant topics were related to the treatment and business-related process (treatment/operational process, disease diagnosis (heart), appointment process, medical examination and patient visit process). In comparison with Stage I, Stage II had new topics related to hospital physical environment (Hospital environment and hospital cafeteria servicescape), treatment process (disease diagnosis (cancer), treatment experience, emergency services and trauma center and chemotherapy (and its side effects) and healthcare provider conduct (doctor professionalism and medical ethics (relational conduct)). The main differences between Stage I and Stage II were that the publics expressed their concerns in their topics regarding disease diagnosis (heart), doctor’s attitude, communication (listen and explain) and appointment process in Stage I, while disease diagnosis (cancer), treatment experience, chemotherapy and its side effects, treatment cost and staff attitude (unfriendly and non-cooperative staff) in Stage II. Patients expressed their dissatisfaction with staff attitude in Stage I, while expressed their satisfaction in Stage II. Topics in Stage III were quite different from the earlier stages due to the COVID-19 disease outbreak. Following the pathogen’s initial detection as a new coronavirus, the public started to look for the new coronavirus information to learn the necessary knowledge. Topics included in this stage included (clinical characterization, virus transmission, travel restrictions, virus symptoms, activities, government early countermeasures, vaccine development/treatment, quarantine measures and effects, gratitude healthcare and reduce spread and materials supply during COVID-19). The clinical characterization, virus transmission and travel restrictions were the most prevalent topics in the early period of Stage III. This was because the COVID-19 outbreak spread globally in this stage. On 20 January, the first case was identified in the U.S., with an increasing number of confirmed cases in successive months. The COVID-19 crisis made citizens and the government aware of the significance of prevention. People started to conscious virus symptoms, avoid unnecessary travel, wear masks and maintain social distancing at public places and governments first-tier responses to major public health crises. Additionally, at this stage, the public focus was diverted to online activities, gratitude to medical staff, material donation and medical service in the main disease hit states of the U.S. Furthermore, the outbreak spread across the U.S., and the public was more concerned about the topics such as virus symptoms, government early countermeasures, quarantine measures and effects and materials supply during COVID-19. Finally, the topics about vaccine development/treatment and gratitude to the healthcare staff were also discussed in the PORs after 30 April.

**Figure 2:**
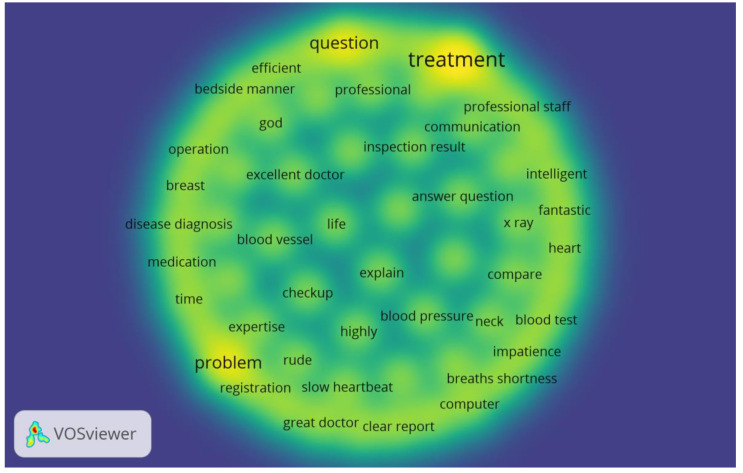
Co-occurrence network and Density visualization network of high-frequency topic keywords in Stage I.

This study also visualized co-occurrence networks of topic keywords across each stage using VOSviewer. As shown in Figure 2, the bigger the node size and font, the greater the keyword’s weight and core location. The co-occurrence network for Stage I data involved the most prevalent topic keywords related to treatment experience with the doctor, questions asked by the patients from their healthcare provider and communication problems between doctors and their patients.

**Figure 3:**
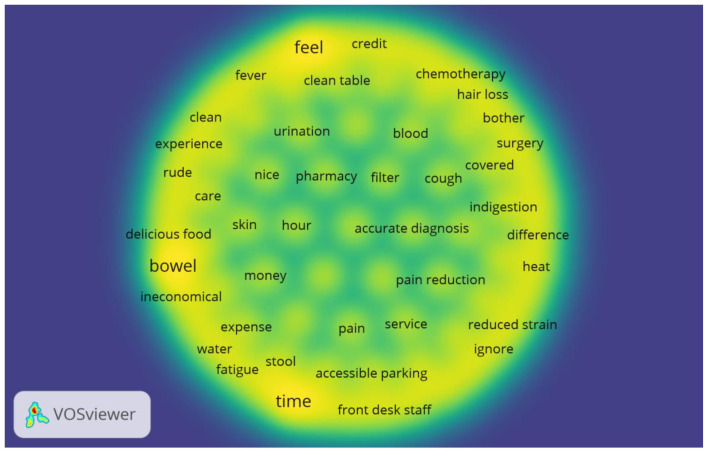
Co-occurrence network and Density visualization network of high-frequency topic keywords in Stage II.

The co-occurrence network for Stage II data involved the prevalent topic keywords such as patients’ feelings in a sound hospital environment, patients involved with cancer diseases going for chemotherapy which resulted most frequently with bowl as its side effects, and physicians spending time with their patients to solve their healthcare problems (see Figure 3).

**Figure 4:**
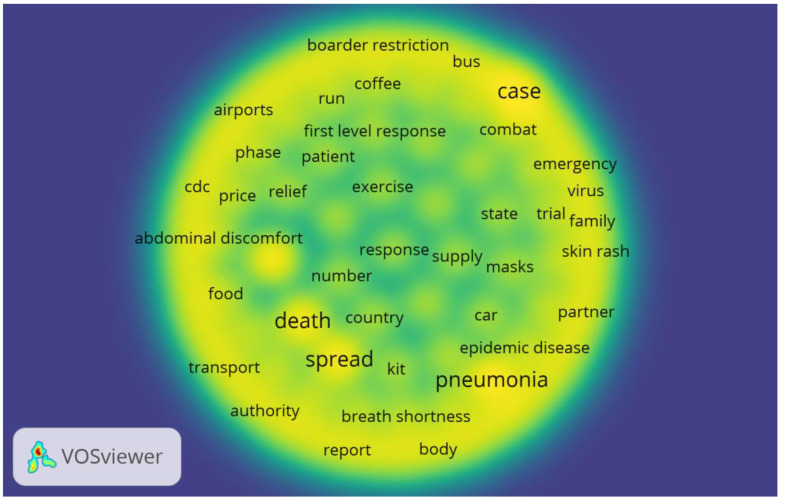
Co-occurrence network and Density visualization network of high-frequency topic keywords in Stage III.

The co-occurrence network for Stage III data included the prevalent topic keywords, such as the fast spread of pneumonia, which causes the emergence of new confirmed cases, increasing the number of deaths (see Figure 4).

### 4.2. Topic Trends

**Figure 5:**
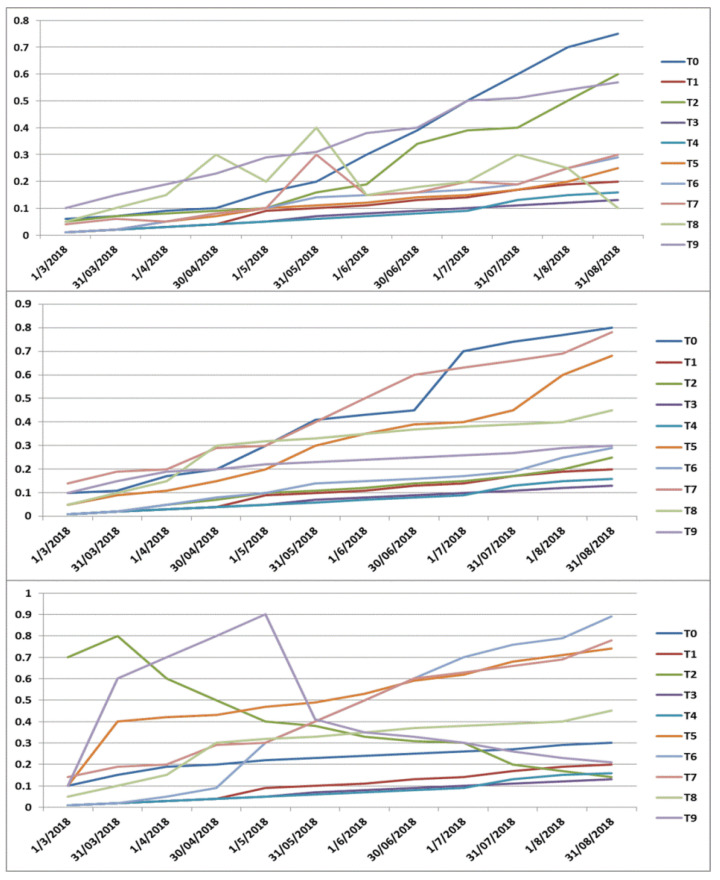
Topic trends change over time in Stages I, II and III.

First, researchers could observe that the online reviews patterns in the 1st and 2nd stage were very identical. While there were minor variations, the overall increase and decrease trends were almost similar. For example, doctor competence, patient visit process and disease diagnosis (heart) were the most discussed topics in Stage I. Additionally, increase and decrease variations were observed in topics, such as doctor value and medical examination across the six months of Stage I data collection (see Figure 5a).

Figure 5b presented the topic trends in PORs for Stage II. Doctor professionalism, hospital servicescape, treatment cost and medical ethics (Relational conduct) were the most prevalent topics at this stage.

Figure 5c showed the topic trends in PORs for Stage III. The topic trends were quite different from the early two stages due to the novel COVID-19 disease outbreak. Topics such as travel restrictions, government early countermeasures, vaccine development/treatment, quarantine measures and effects and materials supply during COVID-19 were the most discussed topics at this stage. For example, travel restrictions (T2) show a peak in March and sharply decreases in the following months. Second, the trend is attributed to public health initiatives. For example, the topic about materials supply during COVID-19 (T9) started to increase till the end of April but decreased drastically due to the balance in the demand and supply of materials. Government early countermeasures (T5) were also emphasized in PORs. In the U.S., many states issued stay-at-home orders around that time as well. Additionally, the number of tests and cases were growing steadily and therefore discussed frequently under this topic. Interestingly, the topics about vaccine development/treatment (T6) and gratitude to the healthcare staff were not frequently discussed till the end of April. But the discussion started drastically due to several companies in China, Russia and the U.S., involved in vaccine development and their clinical trials.

### 4.3. Public Reactions to Different Topics across Three Stages

After performing ABSA, the authors computed the hot topics sentiments across three stages of public discussion in PORs. Emotions have been categorized as positive, negative and neutral. Based on the Plutchik’s Wheel of Emotions [59], positive emotions were further divided into joy, anticipation, surprise and trust. Negative emotions were sub-categorized into anger, sadness, disgust and fear. There was no subdivision for neutral emotions.

**Figure 6:**
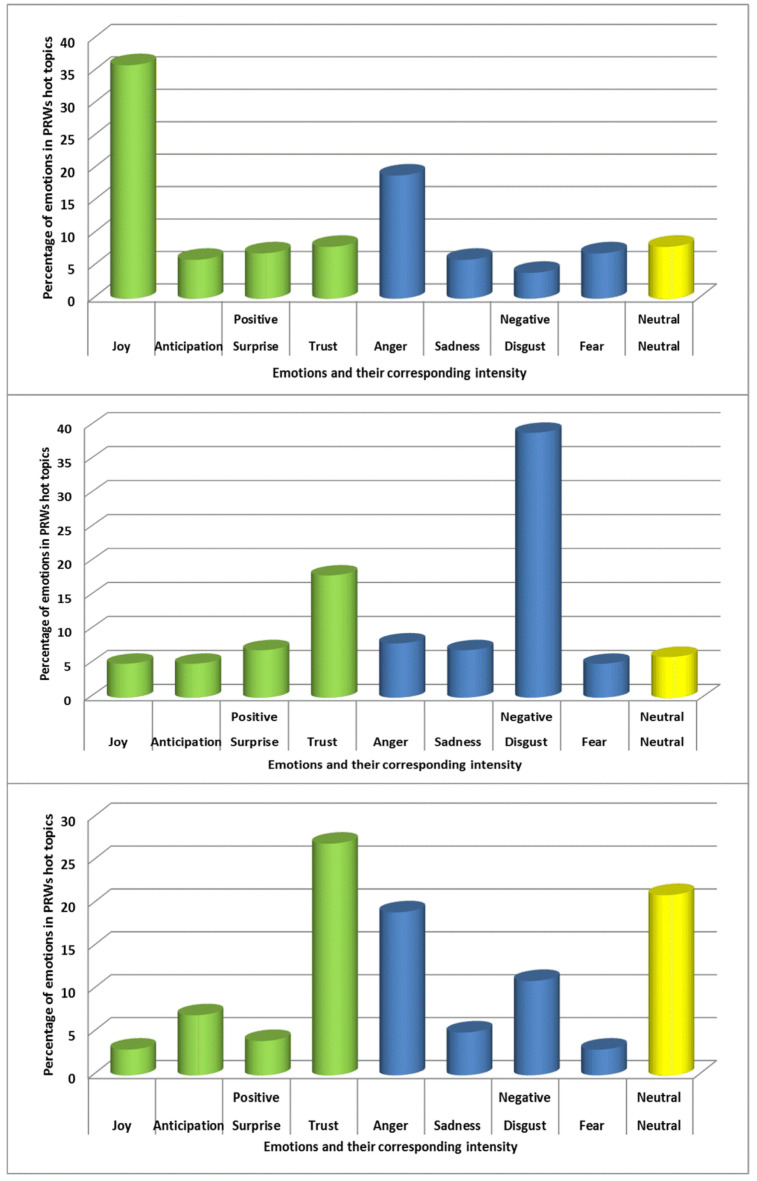
The sentiment statistics of the hot topics on four PRWs in Stage I, II and III.

As shown in Figure 6a, it has been observed that the sentiment of the hot topics of the four PRWs in Stage I tended to be positive, accounting for 56% of positive emotions, of which joy = 35%, anticipation = 6%, surprise = 7% and trust = 8%. Negative emotions accounted for the 36%, of which anger = 19%, sadness = 6%, disgust = 4% and fear = 7%. The neutral emotions accounted for the lowest proportion at 8%.

For Stage II in Figure 6b, it can be found that the sentiment of the hot topics tended to be negative, accounting for 59% of negative emotions, of which anger = 8%, sadness = 7%, disgust = 39% and fear = 5%. Positive emotions accounted for the 35%, of which joy = 5%, anticipation = 5%, surprise = 7% and trust = 18%. The neutral emotions accounted for the lowest proportion at 6%.

For Stage III in Figure 6c, 41% of the hot topics revealed positive emotions, of which joy = 3%, anticipation = 7%, surprise = 4% and trust = 27%. Negative emotions accounted for the 38%, of which anger = 19%, sadness = 5%, disgust = 11% and fear = 3%. The neutral emotions accounted for the lowest proportion at 21%.

Based on the detailed study of three-stage emotional patterns, the general public negative emotions for the COVID-19 outbreak have been diminished, and its positive emotions are generally strengthened.

### 4.4. Performance Comparison Results

**Figure 7:**
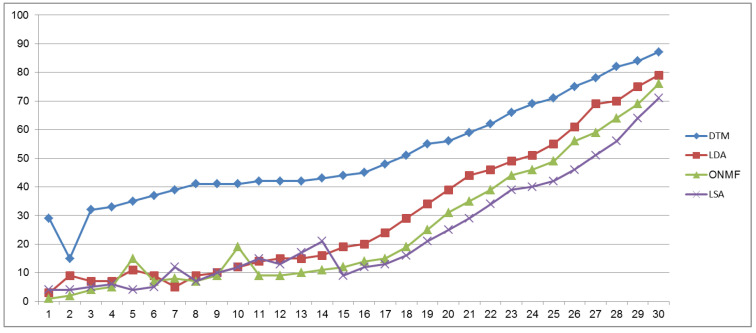
Evaluation of the proposed DTM, LDA, ONMF and LSA models for topic mining and tracking in PORs.

According to Figure 7, the highest accuracy was delivered by the proposed DTM with 30 topics; it outperformed the LDA, non-negative matrix factorization and latent semantic analysis approaches. The experimental results showed that the lower the number of words in a text, the worse the algorithmic output is, and this is because unseen data weaken algorithmic performance. The proposed method, which used the Sentic-LSTM model to classify all PORs into the majority class, achieved accuracy and recalls of 87.12% and 82.35%, respectively, which was significantly higher than the accuracy and recall of other ML algorithms (see Table 1). The authors assume that sentiment and semantic approaches can produce useful outcomes by enabling an overview of how individuals feel about various aspects of healthcare.

## 5. Discussion

In recent years, PRWs have played an important role in evaluating patients’ quality of care as people continually search health information online [60,61,62]. In particular, this study incorporated and expanded developments in the online marketing and healthcare operations literature, thus advancing the authors’ understanding of patients’ preferences in online healthcare markets. This work is supported by the rapid growth of research on social media content. While a growing number of papers in the information systems and marketing domain have analyzed text data to investigate customer behavior, there has been little research into mining healthcare service reviews to investigate patient opinions and attitudes. In contrast, empirical operations management (OM) literature has begun to look into the importance of social media content on operations [63], but limited analysis has exploited the information from the online healthcare industry. In comparison to product reviews, the features for PORs have a more varied style due to the intangibility of the service [11].

When dealing with fast-moving infectious disease outbreaks, measuring the awareness and attitudes of relevant communities must be done quickly if the results are to be useful to the public health response. Traditional surveys usually take several months to prepare, and data collection have experienced increasingly low responses (usually well below 10%), which can be a significant source of bias when substantial weighting changes are produced [64]. Big social data removes the time lag caused by traditional surveys that enable scientists to analyze public opinion on topics in real time and also solve privacy concerns of users by analyzing public activities in groups with specific problems. In particular, public opinion mining has made it easier to learn about people perspectives on important issues [21].

Experts from the Centers for Disease Control and Prevention (CDC) and the World Health Organization (WHO) acknowledge that they mishandled the Ebola response by failing to react to the threat earlier. One year after the Ebola epidemic ended, the Zika virus began to spread, leading to an increase in fear and misinformation. Recently, citizens have become increasingly conscious of their surroundings with the proliferation of smartphones and social media websites like Facebook and Twitter [21]. The concept behind citizen consciousness is that people act as sensors in the environment, providing information about health care issues such as Ebola and Zika virus outbreaks [14]. Regarding Zika, Twitter served as a source of misinformation [21]. This study analyzed PORs datasets from PRWs in the U.S. Website regulators, and healthcare officials monitor the posting of PORs in order to avoid the postings of rumors and misinformation from public. Since previous studies mostly focused on the Twitter data for social media analysis of different pandemics [8,14,21,65], to the authors’ knowledge, there are limited studies in the U.S. context which incorporated social media analytics of public response expressed in PORs across multiple years, including the current period of COVID-19 disease outbreak. Social media research provides this potential because of the extensive usage of these channels and the relative speed at which data can be collected [20].

This work is the first to systematically assess the PORs from four U.S.-based PRWs to analyze the public attention toward different healthcare aspects in three stages (between March and August of 2018, between March and August of 2019 and between March and August of 2020). The current study contributed fourfold. First, based on the dynamic topic modeling, this study identified different topics that demonstrate the public reactions toward healthcare quality and different health interventions. Second, the identified topic keywords across three stages were visualized through social semantic network analysis (i.e., overlay visualization and density visualization). Third, authors have drawn the different topic trends changing over time across three data collection stages using document-to-topic distribution. The topic dynamics in Stage III (i.e., COVID-19) were quite different that from Stage I and Stage II. Fourth, based on the hybrid approach to ABSA, sentic computing framework and Plutchik’s Wheel of Emotions, this study tracked the public emotions toward the hot topics expressed in PORs. The hybrid method offers the depth of understanding provided by qualitative approaches and the measurability of computational techniques.

Expressed sentiments dominate from positive to negative and then positive across the three stages of the study. In sum, negative emotions diminished, and positive emotions soared. In this study, the public reaction and attention to the various aspects of health care are examined, allowing public health practitioners to track public response, identify public needs as soon as possible and take timely public health prevention and control steps. The findings also showed that the use of ML approaches to mine pandemic PORs will give agencies and healthcare providers useful data. For example, this research discovered that sentiments varied by time and that time-variant PORs could help local governments use data to tailor initiatives and communications to community needs. Digital data, including PORs, will incorporate epidemiological data in real-time so that pandemic situations can be analyzed more comprehensively and instantly [66]. This is critical because conventional data on public health can be made accessible within 1 to 2 weeks. Because of their sheer number, PRWs data can also aid in identifying and tracking unusual event events in public, such as the multisystem inflammatory syndrome linked with COVID-19 [9]. Moreover, PRWs provide a low-cost and productive forum for evaluating the efficacy of public health messaging and focusing public health efforts on the prevailing topics of PRW discussion [8]. Using ML to analyze PORs may also reveal how the public interprets mixed signals about various healthcare issues.

From 1 March 2018 to 31 August 2020, the public focused regarding various healthcare aspects on PRWs can be divided into three stages. In the first stage (1 March 2018, to 31 August 2018), the U.S. public expressed their satisfaction toward doctor competence, treatment/operational process, friendly staff, doctor value, medical examination and patient visit process. In contrast, the public was unhappy with disease diagnosis (heart), doctor’s attitude, communication (listen and explain), the appointment process and patient visit process.

In the second stage (1 March 2019, to 31 August 2019), the U.S. public expressed their satisfaction toward doctor professionalism, disease diagnosis (cancer), emergency services and trauma center, hospital cafeteria servicescape, hospital environment and medical ethics (relational conduct). In contrast, the public showed their concerns toward disease diagnosis (cancer), treatment experience, chemotherapy and its side effects, treatment cost and unfriendly and non-cooperative staff.

In the third stage (1 March 2020, to 31 August 2020), the U.S. public paid less attention to the COVID-19 because the pathogen was only reported in Wuhan, China.

Compared with the qualitative investigations, it is worth exploring public interest using keyword frequency analysis, as this method is more reliable and trustworthy in the hot topics research and their growth patterns [67]. Based on the high-frequency PORs-based keyword analysis, knowledge about the issues and opinions of PRW users can be collected at different stages. Studies have shown that people interest in various risk diseases on social media has been related to recent news and global events [26].

Regarding hot topic trends, similar trends were observed at Stages I and II. For instance, topics about doctor competence, patient visit process and disease diagnosis (heart) were the most discussed topics in Stage I; while for Stage II, doctor professionalism, hospital environment, treatment cost and medical ethics (relational conduct) were the most prevalent topics at this stage. Studies have found that people will pay attention to and search for prior patients’ treatment experiences while making their healthcare decisions [68].

On 20 January 2020, the first confirmed case was reported in Snohomish County, Washington, and body temperatures began to be measured at the railway stations, bus stops and international airports in the U.S. People started to observe the seriousness of the outbreak, and the degree of attention paid to the COVID-19 outbreak began to increase until 20 April 2020. Due to the rapid spread of the pathogen across the country, the public reacted promptly to information about the COVID-19 outbreak on different online platforms [28]. The reason behind this argument was because the COVID-19 was a novel pneumonia, so no effective medication and vaccine had yet been developed. At the beginning of the disease outbreak, the public was eagerly looking for the important knowledge and online information to maintain their safety, protection, avoid unnecessarily traveling, preferred limited but online activities, government early measures to stop spreading virus and materials supply in the market including masks, hand sanitizers and food [1,29].

People were discussing issues related to health care and the politics intersecting now. The need for assistance from government was related to vital materials, such as personal shielding kit and intensive care services. Health workers spoke about frontline protection as a problem that has adversely affected their physical and mental health [69]. These results were linked to health-care settings in COVID-19 hotspots that had experienced significant resource redistribution [69].

The shutdown’s effect on the U.S. and global economies has been incredibly distressing. Millions of people have lost work and the number of unemployed has risen drastically. The word “week” was a common tern, and it was related to discussions about “jobs”; the words “suspension”, “temporarily” and “strictly” were also mentioned. This result reveals that week and job issues were predominant in the discussions. These results show that, while most conversations revolved around the week and job issues, not all of them included talk about work loss. Some PORs expressed a preference for working from home. This finding indicates that strategies for reopening the economy that include work-from-home opportunities may be both common and effective at reducing virus spread [65].

Having the support of healthcare professionals and peers during high stress can help mitigate the negative effects [70]. On the other hand, social isolation and distancing can make it difficult to receive the necessary support. The study findings revealed that “gratitude” was a hot topic of conversation, and it was specifically related to “family”, “together”, “healthcare staff” and “support”. Despite the isolation, people can find convenience and support through social media connections to their family and friends and soon have time together while staying confident.

After 30 April 2020, even though the number of COVID-19 cases has continued to increase, the discussion topics focused was shifted to research and development of vaccine and gratitude to the healthcare staff for their tireless efforts during the pandemic. This may be due to relatively saturated epidemic information. The disease could also not be taken closely into account by citizens to obtain security over time, and the public knowledge appears to be rational [71]. However, as the probability of a new coronavirus vaccine rises, one concern is that, despite the proven value of vaccines, only about half of the population will choose to receive one. Even a clinically validated vaccine requires widespread acceptance [72] and unfounded fears of negative side effects can outweigh the advantages of coronavirus vaccination. PRWs provide an opportunity to track vaccine acceptance and tailor responses to vaccine opponents. The indigenous risk of vaccine-preventable diseases could increase when geographically grouped public refusing to vaccinate and express more negative sentiments. Social media analytics offers a potentially useful and economical method to officials in the field of public health to identify and measure the efficacy of regional clusters of interventions.

Sentiment analysis can help to understand how people feel about an event. Based on the sentiment patterns of the hot topics associated with PORs on PRWs, the first stage of emotion tended to be positive, the second stage was negative and the third stage was again positive. On the whole, negative emotions weakened and positive emotions increased. Early investigations have shown that emotions and social media content are very closely related to each other. In Stage I, public attention was mostly focused on the different aspects of healthcare provider service quality. Most of the topics were related to the doctor competence, friendly staff, doctor value and patient visit process, and emotions lean toward positive. Furthermore, joy and anger were found the leading emotions regarding the discussion topics at this stage.

In Stage II, public concerns were shown in topics such as disease diagnosis (cancer), treatment experience, chemotherapy and its side effects, treatment cost and unfriendly and non-cooperative staff. At that time, there was a strong demand from the public for more information on various aspects of service quality. When the information demand could not be completely fulfilled, the emotions of the users were negative [73]. In addition, trust and disgust were the dominant emotions regarding the discussion topics, but the disgust percentage was higher than the trust.

In Stage III, with the few months after the initial spread of the COVID-19 pathogen, public opinion appeared to be optimistic because more and more news was being published at this stage and the conventional information on PRWs became factual events. The public shrunk prior doubts and worries about the outbreak diminished negative feelings and increased positive emotions. More hot topics included preventive or safety knowledge that encourages and promotes public health communication. In this stage, anger and disgust were the most prevalent emotions in the early period of the disease outbreak due to low-quality information transmission. With the passage of time, particularly after April 30th, the negative emotions were transformed into positive emotions with trust as a more dominant emotion dimension. This transformation was due to the government’s countermeasures for disease control and prevention, vaccine preparation, maintaining the balance between the demand and supply of goods in the market and the healthcare staff’s tireless efforts. Overall, sentiments about the pandemic conveyed in PORs were more likely to be optimistic, which meant the public remained confident in an unexpected public health crisis. Positive opinion keywords often conveyed appreciation for frontline staff and community efforts to help stranded community members. The high percentage of positive sentiments exhibits that public may have miscalculated the seriousness of COVID-19 during the outbreak. Highly impact-oriented strategies could be essential to keep the public involved and confident about the future.

There are certain limitations to this study. First, the authors limited the analysis to English-language PORs only, which means that non-anglophones were not considered. Hence, there could be a major bias in favor of English PORs in the study dataset. Future research could be performed on multilingual data from other PRWs belong to different countries. Second, the current study performed longitudinal topic modeling by comparing topic trends over multiple years. Future research could also analyze and compare the PORs posted on PRWs during the Trump administration or the post-COVID-19 or future potential pandemics scenarios to better comprehend potential temporal patterns. With regards to future work, the authors expect to assess other online media platforms, including Twitter and Facebook, using advanced analytical techniques for retrieving meaningful topics from public comments and classification of sentiments in these topics.

## 6. Conclusions

Public health officials and the media in the U.S. need to understand the general public perceptions and attitudes toward COVID-19 in order to design successful awareness campaigns. This study showed a systematic view of the present research in response to the PORs posted on PRWs. This study found that PRWs can be useful to measure public attention, particularly during a crisis. The current study identified ten hot topics (at each stage) with their corresponding keywords based on the data collection in three different periods. The sentiments expressed in these topics explained the public response to PORs, especially in the COVID-19 stage, which could help government representatives, private agencies and the public with pandemic information. The findings of the current study suggest that public health officials should consider improving particular communication networks, such as social media channels, to balance the typical phenomenon of devotion capacity. In reality, these channels can create more lasting relationships with people through a constant series of direct interactions. Recently, the CDC public health officials are updating people with regular announcements from their Twitter account.

## Figures and Tables

**Table 1:** Performance of the system using PORs datasets.

Models	Accuracy	Recall
SVM	74.32%	69.18%
LSTM	81.45%	78.25%
Sentic-LSTM	87.12%	82.35%

## Data Availability

Not Applicable.

## Appendix A

**Table A1:** Topics and their relevant keywords identified by Topic modeling and manual annotation.

	2018	2019	2020
Topic 0			
	**Doctor competence**	**Doctor professionalism**	**Clinical characterization**
0	Professional	Professional	Respiratory
1	Experienced	Knowledgeable	Pneumonia
2	knowledgeable	Intelligent	Novel coronavirus
3	Skills	Caring	Unknown cause
4	Intelligent	Sincerely	Person-person spread
5	Honest	Help	Death
6	Expertise	Opportunity	Case
7	Listen	Once again	Patient
8	Answering questions	Accurate diagnosis	Epidemic disease
9	Treatment	Serious and responsible	Leave hospital
Topic 1			
	**Treatment/operational process**	**Disease diagnosis (Cancer)**	**Virus transmission**
0	Heart	Cough	Confirmed diagnosis
1	Breast	Bowel	Masks
2	Diagnosis	Blood	Startup
3	Treatment	Stool	First case
4	Neck	Urination	First-level response
5	Changed the life	Testicles lumps	Goods and materials
6	Medical procedures	Indigestion	Nationwide
7	Physical therapy	Headache	Emergency
8	Surgery	Pain	Pneumonia
9	Medication	Fever	Isolation centers
Topic 2			
	**Disease diagnosis (heart)**	**Treatment experience**	**Travel restrictions**
0	Treatment	Problem diagnosis	Suspension
1	Surgery	Bad	Operation
2	Pain	Worst experience	Transport
3	Long time	Wrong hospital	Temporarily
4	Blood pressure	Painful	Strictly
5	Blood vessel	Long wait time	Airports
6	Disease diagnosis	Wrong	Boarder restrictions
7	Dizziness	Experience	Bus stands
8	Breaths shortness	Years	Long-distance
9	Slow heartbeat	Slow recovery	Spread
Topic 3			
	**Doctor’s attitude**	**Emergency services and trauma center**	**Virus symptoms**
0	Rude	24 h	Fever
1	Depressed	Pharmacy	Cough
2	Long	dressing	Headache
3	Wait	Pain reduction	Sore throat
4	Questions	Medical doctor	Skin rash
5	Busy	Surgery	Fatigue
6	Careless	Reduced strain	Body aches
7	Less time	Trauma	Abdominal discomfort
8	Check	Special facilities	Stuffy nose
9	Ignore	Specialized equipment	Breath shortness
Topic 4			
	**Friendly staff**	**Hospital cafeteria servicescape**	**Activities**
0	Helpful	Hygienic	Home schooling
1	Friendly	Clean	School canceled
2	Amazing	Clean table	Online teaching
3	Wonderful	Delicious food	Week
4	Fantastic	Covered	Run
5	Bedside manner	Filter	Park
6	Efficient	Water	Exercise
7	Ease	Quick service	Diet
8	Professional staff	Fresh food	Job
9	Great staff	Comfortable environment	Coffee
Topic 5			
	**Communication (listen and explain)**	**Hospital environment**	**Government early** **countermeasures**
0	Bad	Office	WHO
1	Problem	Fantastic	Infectious disease
2	Compare	Clean	Disease management
3	Uncomfortable	Accessible parking	Authorities
4	Communication	Comfortable	Big move
5	Impatience	Sitting room	CDC
6	Worry	Nice	Testing procedures
7	Poor explanation	hospital cafeteria	Response
8	Nervous	Feel	Ventilators
9	Unhappy	Comfortable	Testing kits
Topic 6			
	**Appointment process**	**Chemotherapy and its side effects**	**Vaccine development/treatment**
0	Registration	Painful	Launch
1	Wait	Side effect	Trial
2	Hours	Heat	Partner
3	Queue	Chemotherapy	First
4	Online system failure	Bowel	Combat
5	System performance	Hair loss	Clinical
6	Computers	Skin	Phase
7	Staff absence	Nail problems	Researcher
8	Bad	Fatigue	Relief
9	Long	Nausea	Genetic
Topic 7			
	**Doctor value**	**Treatment cost**	**Quarantine measures and effects**
0	Recommend	Expenses	lockdown
1	Highly recommend	Money	Virus
2	Kind caring	Registration fee	Death
3	Excellent doctor	Waste money	Spread
4	Great doctor	Expensive	Test
5	Lovedr	Ineconomical	Number
6	God	Affordability	Follow
7	Trust	Difference	Report
8	Pleased	Insurance	State
9	Impressed	Credit	Country
Topic 8			
	**Medical examination**	**Medical ethics (Relational conduct)**	**Gratitude healthcare** **and reduce spread**
0	Operation	Knowledgeable	Thank
1	X-ray	Great	Together
2	Blood test	Care	Stay safe
3	Advice	Family	Help
4	Clear report	Manner	Maintain
5	Prescription	Wonderful	Protect
6	Inspection result	Feel	Donate
7	Problem	Recommend	Washingyourhands
8	Reexamine	Excellent	Family
9	Checkup	Time	Follow
Topic 9			
	**Patient visit process**	**Unfriendly and non-cooperative staff**	**Materials supply during COVID-19**
0	Takes time	Staff	Food
1	Answer questions	Unfriendly	Price
2	Explain	Rude	Sanitizer
3	Listen	Front desk staff	Control
4	Attentive	Call	Market
5	Compassionate	Unhelpful	Production
6	Responsive	Ignore	Demand
7	Friendly	Loud	Supply
8	Cooperative	Twice	Manufacture
9	Help	Bother	Surgical equipment

**Note:** Topic labels in bold.
